# NVD-BM-mediated genetic biosensor triggers accumulation of 7-dehydrocholesterol and inhibits melanoma via Akt1/NF-ĸB signaling

**DOI:** 10.18632/aging.103562

**Published:** 2020-07-25

**Authors:** Jia Liu, Lei Cao, Jun-Ze Qu, Ting-Ting Chen, Zi-Jie Su, Yun-Long Hu, Ying Wang, Ming-Dong Yao, Wen-Hai Xiao, Chun Li, Bo Li, Ying-Jin Yuan

**Affiliations:** 1 Frontier Science Center for Synthetic Biology and Key Laboratory of Systems Bioengineering, Ministry of Education, Tianjin University, Tianjin 300072, China; 2 Collaborative Innovation Center of Chemical Science and Engineering (Tianjin), School of Chemical Engineering and Technology, Tianjin University, Tianjin 300072, China; 3 Guangdong Key Laboratory for Genome Stability and Disease Prevention, Carson International Cancer Center, Department of Pharmacology, Shenzhen University Health Science Center, Shenzhen 518060, China; 4 Guangdong Provincial Key Laboratory of Regional Immunity and Diseases, Department of Pathogen Biology, Shenzhen University, Health Science Center, Shenzhen 518055, China

**Keywords:** melanoma, NVD-BM, cholesterol 7-desaturase, genetic biosensor, cancer cell regression

## Abstract

Aberrant activation of the cholesterol biosynthesis supports tumor cell growth. In recent years, significant progress has been made by targeting rate-limiting enzymes in cholesterol biosynthesis pathways to prevent carcinogenesis. However, precise mechanisms behind cholesterol degradation in cancer cells have not been comprehensively investigated. Here, we report that codon optimization of the orthologous cholesterol 7-desaturase, NVD-BM from *Bombyx mori*, significantly slowed melanoma cell proliferation and migration, and inhibited cancer cell engraftment in nude mice, by converting cholesterol to toxic 7-dehydrocholesterol. Based on these observations, we established a synthetic genetic circuit to induce melanoma cell regression by sensing tumor specific signals in melanoma cells. The dual-input signals, RELA proto-oncogene (RELA) and signal transducer and activator of transcription 1 (STAT1), activated NVD-BM expression and repressed melanoma cell proliferation and migration. Mechanically, we observed that NVD-BM decreased Akt1-ser473 phosphorylation and inhibited cytoplasmic RELA translocation. Taken together, NVD-BM was identified as a tumor suppressor in malignant melanoma, and we established a dual-input biosensor to promote cancer cell regression, via Akt1/NF-κB signaling. Our results demonstrate the potential therapeutic effects of cholesterol 7-desaturase in melanoma metabolism, and provides insights for genetic circuits targeting 7-dehydrocholesterol accumulation in tumors.

## INTRODUCTION

Globally, melanoma is a serious malignant skin cancer, and is estimated to kill approximately 55,500 individuals annually [1]. Increasing melanoma incidences are reported in older Caucasians, with males experiencing higher rates than females [2, 3]. Traditional surgical and chemo-radiotherapy treatments often fail to prolong patient survival, especially in those with advanced disease. This is due to metastasis and chemotherapy resistance, primarily due to acquired somatic mutations, e.g., B-Raf oncogene serine/threonine-kinase (BRAF), NRAS proto-oncogene (NRAS) and neurofibromin 1 (NF1) [4]. Targeted therapies such as dabrafenib and vemurafenib are proven melanoma treatments, generating positive results in clinical settings [5–9]. However, drug resistance after a period of treatment, leads to poor responses or relapses in advanced melanoma patients [10, 11]. Recent studies have demonstrated that metabolic reprogramming plays important roles in melanoma tumorigenesis, and may be responsible for drug resistance and poor prognoses [12]. Therefore, the identification of novel therapeutic strategies targeting cancer metabolism, and the concomitant development of therapeutic drugs are essential for melanoma cancer therapy.

Cellular cholesterol levels are tightly regulated by an intricate network of transcriptional regulation of cholesterol biosynthesis, deposition, cellular uptake and efflux programs [13–15]. Cholesterol metabolism dysregulation is associated with cancers. Oncogenic processes permits cholesterol synthesis in cancer cells, which is further metabolized to support rapid proliferation [16]. Recent investigations have shown that cholesterol biosynthesis gene activation is associated with poor prognoses in leukemia, sarcoma and melanoma [17]. Indeed, perturbed expression of key cholesterol biosynthesis enzymes, such as HMGCR and SQLE, prevents carcinogenesis in colorectal cancer, breast cancer, prostate cancer and hepatocellular carcinoma associated with nonalcoholic fatty liver disease (NAFLD-HCC) [18–22]. However, the role of cholesterol and its metabolites in cancer is still controversial [17, 23–25]. In breast cancer, decreased CYP7B1 expression triggers 27-hydroxycholesterol (27HC) accumulation [26], permitting 27HC to act as an estrogen receptor agonist that induces tumor growth and metastasis [25]. Other reports have observed that the cholesterol metabolites, dendrogenin A [27], cholestane-3β, 5α and 6β-triol [28] exert anti-proliferative effects in human breast and prostate cancer cells [28, 29]. Of note, the cholesterol metabolite, 7-dehydrocholesterol (7-DHC) exhibits toxic and pro-apoptotic effects towards melanoma cells [30], thereby encouraging us to develop a therapeutic strategy incorporating cholesterol conversion to 7-DHC.

Although the cholesterol 7-desaturase conversion of cholesterol to 7-DHC has not yet been reported in mammalian cells, several evolutionary conserved isoenzymes exist in orthologous organisms, such as NVD-BM from *Bombyx mori*, DAF-36 from *Caenorhabditis elegans*, NVD-DR from *Danio rerio*, TTHERM_00310640 from *Tetrahymena thermophila SB210* and MGC154819 from *Xenopus laevis* [31–33]. Natural products including 7-DHC have been proven in cancer therapy, however, dose administration and side effects often hinder their clinical applications [34]. Designing and synthesizing genetic regulatory circuits in living cells, to program new biological behaviors, is a promising synthetic biological approach for medicinal research and potential therapeutic applications [35, 36]. Reprogrammed human cells can circumvent the shortcomings of conventional therapies, such as frequent administration and self-sufficient doses, providing new strategies to solve the complexities of metabolic disorders [37]. In this study, we performed codon optimization on several cholesterol 7-desaturases to examine their roles in melanoma cell proliferation, migration and tumorigenicity, both *in vitro* and *in vivo*. Furthermore, we developed a smart genetic circuit to induce 7-DHC accumulation by sensing transcription factors in melanoma cells.

## RESULTS

### Cholesterol 7-desaturase expression inhibits melanoma cell growth and migration

**Figure 1:**
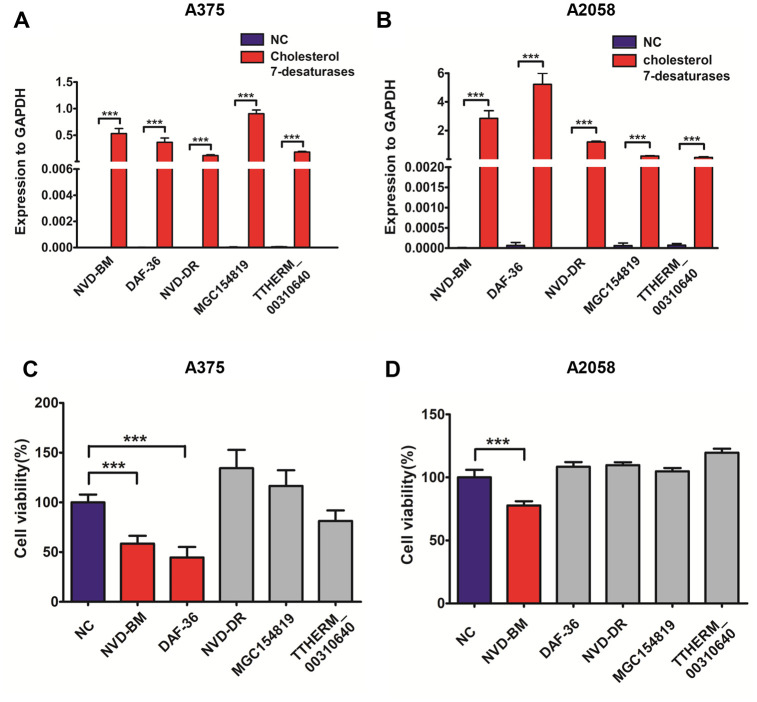
**Heterogeneous cholesterol 7-desaturases inhibits melanoma cell growth.** (**A**, **B**) Quantification of GAPDH, NVD-BM, DAF-36, NVD-DR, MGC154819 and TTHERM_00310640 gene expression using RT-qPCR in A375 and A2058 cells. (**C**, **D**) A375 and A2058 cell proliferation transfected with genes expressing different cholesterol 7-desaturases at 24 h. (***p < 0.001)

**Figure 2:**
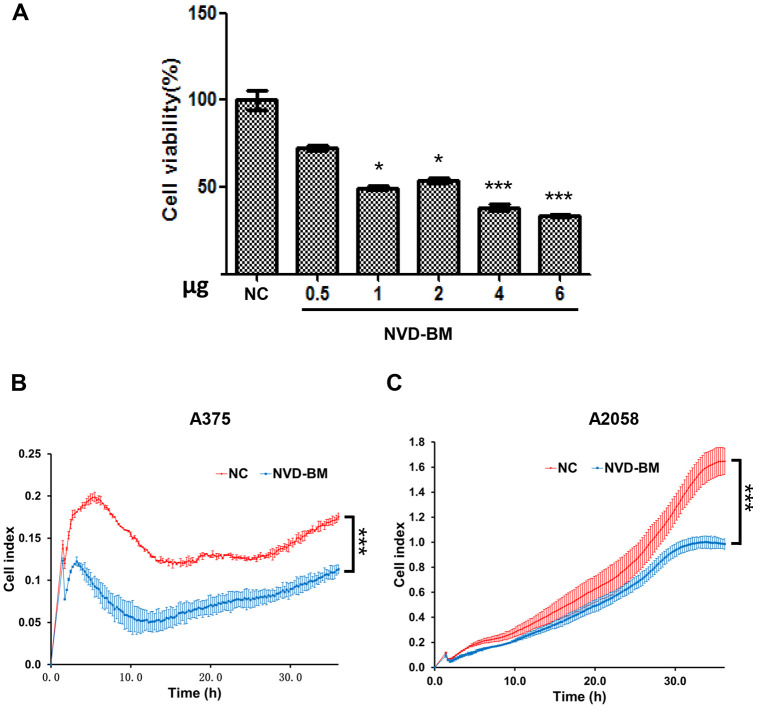
**NVD-BM inhibits melanoma cell proliferation and migration.** (**A**) A375 cell proliferation when transfected with different NVD-BM concentrations at 24 h. (**B**, **C**) The migration rates of 5×10^3^ A375 and A2058 cells, transfected with pCDH and NVD-BM plasmid were assessed over 36 h by RTCA assay. (*p < 0.05, **p < 0.01, ***p < 0.001)

Since no cholesterol 7-desaturases exists in mammalian cells, we optimized codon usage and synthesized several cholesterol 7-desaturases from other species; DAF-36 from *Caenorhabditis elegans*, NVD-BM from *Bombyx mori*, NVD-DR from *Danio rerio* (zebrafish), TTHERM_00310640 from *Tetrahymena thermophila SB210* and MGC154819 from *Xenopus laevis* (Table 1, Supplementary Table 1)*.* This approach facilitated anti-cancer studies in melanoma cells. Using transient transfection, the five humanized cholesterol 7-desaturases were expressed in A375 and A2058 melanoma cells by qRT-PCR assay (Figure 1A, 1B). Cell proliferation assays showed that the expression of NVD-BM significantly slowed cell growth of melanoma cells (A375 and A2058), however, DAF-36 failed to suppress A2058 cell proliferation and other cholesterol 7-desaturases did not inhibit melanoma cell proliferation (Figure 1C, 1D). Furthermore, the proliferation effects exerted by NVD-BM expression were dose dependent in A375 cells (Figure 2A). We next investigated cell migration capabilities in cells expressing NVD-BM, using the real-time cell analysis (RTCA). These data showed that A375-NVD-BM and A2058-NVD-BM cell migration capabilities were lower than the empty vector negative control (NC) group at 36 h, suggesting that NVD-BM severely impaired melanoma cell migration (Figure 2B, 2C).

**Table 1:** Heterogeneous cholesterol 7-desaturase.

**Gene Symbol**	**Annotation**	**Species**	**Gene ID**
DAF-36	Cholesterol 7-desaturase	*Caenorhabditis elegans*	179422
NVD-BM	Rieske-domain protein Neverland	*Bombyx mori*	733067
NVD-DR	Neverland	*Danio rerio*	436885
TTHERM_00310640	Rieske-like [2Fe-2S] domain protein	*Tetrahymena thermophila SB210*	7840976
MGC154819	MGC154819 protein	*Xenopus laevis*	779348

### NVD-BM inhibits melanoma cell proliferation in xenograft mice

**Figure 3:**
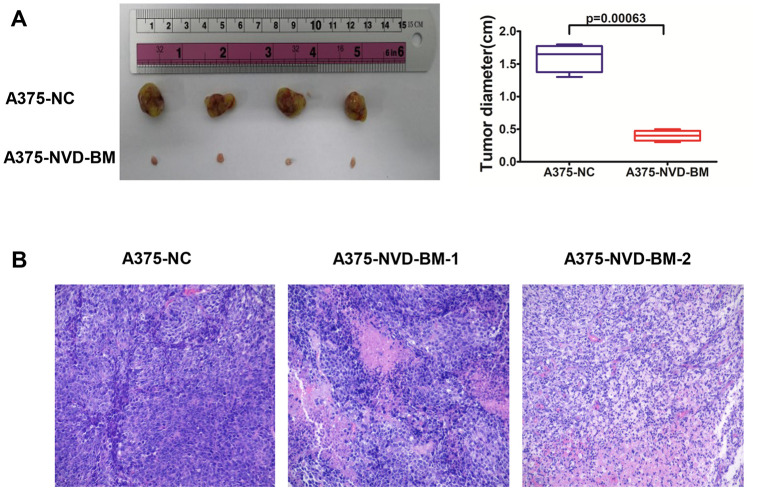
**NVD-BM inhibits melanoma proliferation in xenograft mice.** (**A**) Subcutaneous tumors generated in BALB/c-nu/nu mice, with NVD-BM transduced A375 cells. A375 cells transduced with blank lentiviral vector, and vectors expressing pCDH and NVD-BM were collected and subcutaneously injected into BALB/c-nu/nu mice, at a cell density of 1×10^7^ to elicit tumorigenicity. Tumors were removed and diameters measured (n = 4/group) ± standard deviation (SD). (**B**) Hematoxylin eosin (HE) tumor staining in transduced A375 cells. Nuclei are blue. Cytoplasm is red. Magnification × 200.

The proliferation and migration inhibitory capabilities of NVD-BM in melanoma cells suggested anti-tumor effects *in vitro*, therefore we sought to recapitulate these effects *in vivo.* To confirm the anti-tumor effects of NVD-BM *in vivo*, A375 cells expressing NVD-BM and the empty vector NC, were subcutaneously injected into BALB/c-nu/nu mice (n = 4). Mice were sacrificed at the end of the fourth week, with tumors excised, measured and stained with hematoxylin-eosin (HE). Our data demonstrated that tumor diameter from A375 cells expressing NVD-BM, were approximately four folds smaller significantly than the NC group (p = 0.00063) (Figure 3A). Moreover, overall tumor structures were almost normal, and displayed no obvious necrosis (Figure 3B). In contrast, the NC group displayed large areas of necrosis, nuclear degradation and cytoplasmic protein degradation. These data demonstrated that NVD-BM served as an effective tumor suppressor in melanoma derived cells *in vivo.*

### NVD-BM catalyzes cholesterol to 7-DHC in melanoma cells

**Figure 4:**
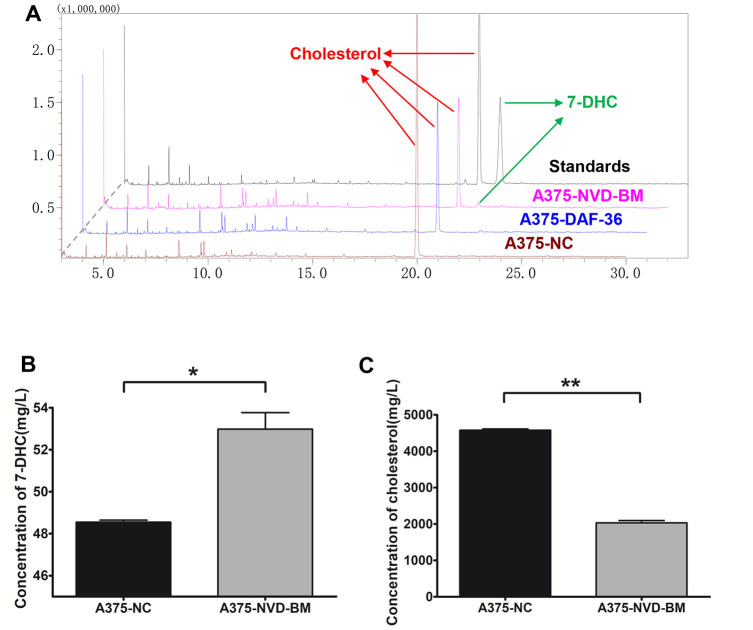
**Heterogeneous cholesterol 7-desaturase NVD-BM converts cholesterol to 7-DHC.** (**A**) GC/MS analysis of 7-DHC and cholesterol levels in A375 cells, transfected with NVD-BM and DAF-36, when compared with the negative control pCDH (NC), using 7-DHC and cholesterol standard curves. (**B**) 7-DHC concentrations in A375 cells, transfected with NVD-BM versus NC. (**C**) Cholesterol concentrations in A375 cells, transfected with NVD-BM versus NC. (*p < 0.05, **p < 0.01).

To test cholesterol 7-desaturase capability in converting cholesterol to 7-DHC, we extracted sterols from A375 cells with the expression of cholesterol 7-desaturases, and determined cholesterol and 7-DHC concentrations using GC-MS (Figure 4A). Our data revealed that NVD-BM converted cholesterol to 7-DHC, and was 2.1 folds compared with NC group (Figure 4B, 4C). DAF-36 failed to convert cholesterol to 7-DHC in A375 cells, suggesting that DAF-36 inhibited A375 cell proliferation via alternative, non-cholesterol mediated mechanisms. Therefore, NVD-BM appeared to effectively inhibit melanoma growth via cholesterol degradation, to increase 7-DHC levels in cells. This observation was consistent with the previous report showing that the proliferation of melanoma cells was inhibited by 7-DHC addition (Supplementary Figure 1).

### The development of a dual-input signal genetic biosensor

**Figure 5:**
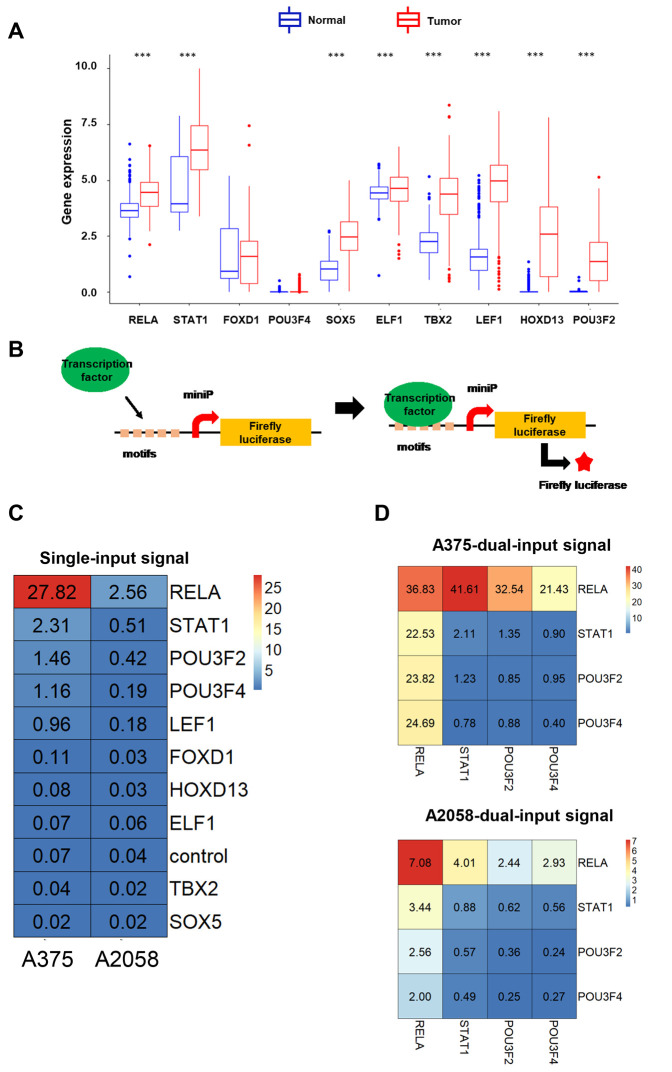
**The development of dual-input biosensors.** (**A**) Up-regulated transcription factors in melanoma tissues compared with normal skin tissues, were filtered from the TCGA database. (**B**) A schematic diagram of transcription factors as input signals, activating biosensors using the luciferase assay. (**C**) Heatmap of luciferase expression levels using a single-input promoter in A375 and A2058 cells. (**D**) Heatmap of luciferase expression levels using dual-input promoters in A375 and A2058 cells. (***p < 0.001).

To enhance the efficacy and specificity of NVD-BM, based on a genetic circuit, we were ought to scan highly expressed transcription factors as dual-input sensors. We compared gene expression profiling data from melanoma tissue with normal skin tissue, and identified several differentially expressed melanoma genes. The top 10 highly expressed transcription factors, RELA, STAT1, FOXD1, POU3F4, SOX5, ELF1, TBX2, LEF1, HOXD13 and POU3F2, were then evaluated for their ability to drive luciferase expression (Figure 5A). Five repetitive transcription factor binding motifs were separately cloned into firefly luciferase vectors, driven by mini-promoters, to determine firefly luciferase expression in A375 and A2058 cells (Supplementary Table 2, Figure 5B). As shown in Figure 5C, RELA, STAT1, POU3F2 and POU3F4 were the top four input signals in A375 and A2058 cells. Next, vectors with different input signals, were divided into 16 permutation methods. Heatmaps showed that the optimum match was RELA and STAT1 in A375 and A2058 cells, respectively (Figure 5D). Finally, RELA and STAT1 were selected as dual-input signals for a genetic biosensor in melanoma cells.

### The activated genetic biosensor inhibits melanoma cell growth and migration

**Figure 6:**
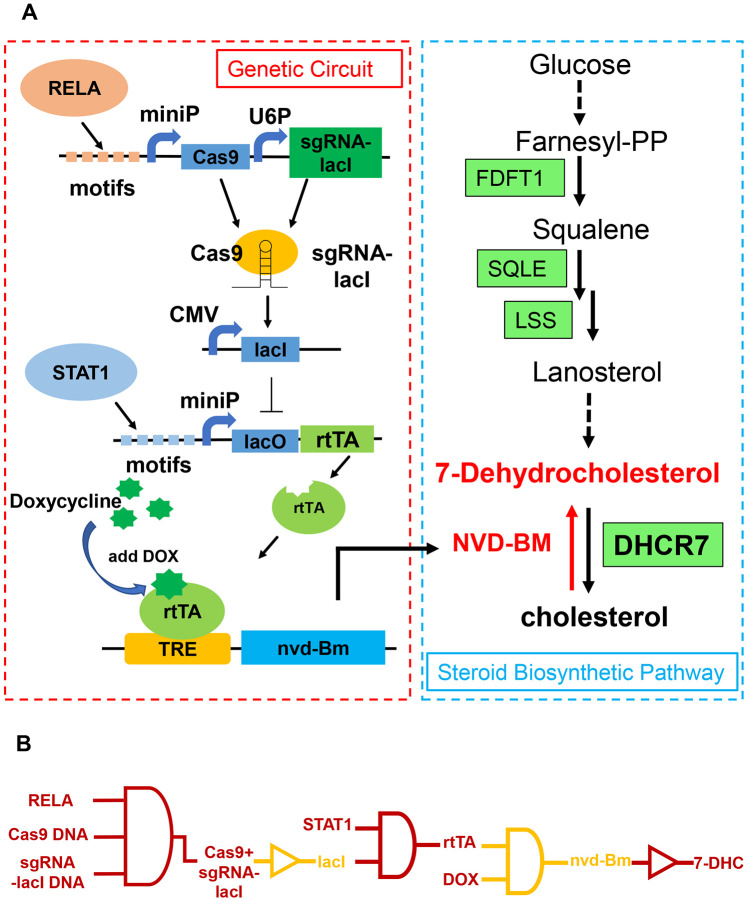
**Schematic of a genetic circuit in melanoma.** (**A**) A gene circuit, with four modules was activated by two up-regulated transcription factors (RELA and STAT1), to express cholesterol 7-desaturase NVD-BM, and directly catalyzing cholesterol to 7-DHC, to repress melanoma growth and migration. (**B**) Gene circuit diagram constructed in melanoma cells based on CRISPR/Cas9 and Tet-ON systems.

We next developed a molecular switch combined with CRISPR-Cas9 and TET-ON, with four modules to activate NVD-BM expression and inhibit melanoma cell growth and decrease migration rates (Figure 6A). Five repetitive motifs from RELA (5’- GGGAATTTCC-3’), Cas9 and sgRNA, targeting the lacI gene were assembled into one construct, facilitating RELA binding to induce Cas9 expression. The Cas9 protein with sgRNA-lacI in the next module, inhibited lacI expression. Five repetitive motifs from STAT1 (5’- CATTTCCCGGAAACC-3’), lacO and rtTA were constructed in one vector, recruiting STAT1 binding to induce rtTA expression. In case of toxicity, NVD-BM was not expressed without doxycycline. When doxycycline was added to the culture medium, it entered cells and bound rtTA. Then, rtTA and doxycycline bound to the TRE gene to activate NVD-BM expression. Thus, when RELA and STAT1 were up-regulated in a tumor, parallel to doxycycline added *in vitro*, the genetic circuit was then switched on (Figure 6B).

**Figure 7:**
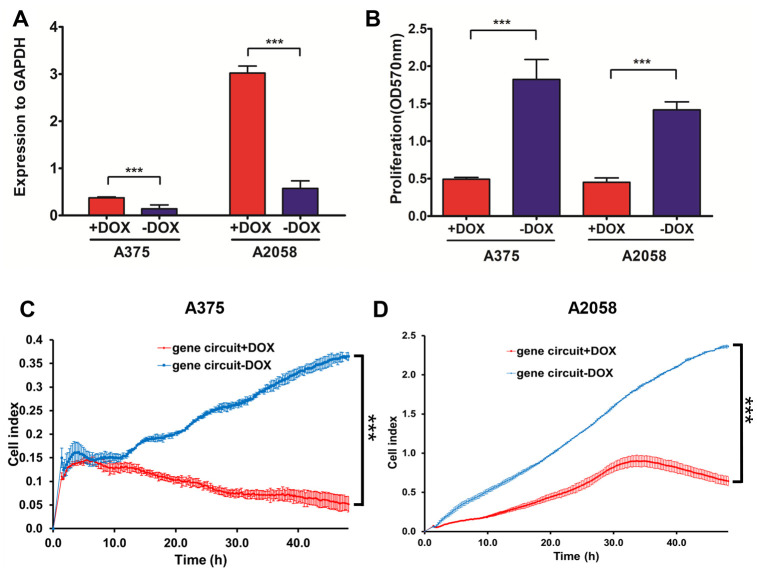
**The activated genetic circuit inhibits melanoma cell growth and migration.** (**A**) A375 and A2058 cells were co-transfected with genetic circuit plasmids. The relative expression of the reporter gene NVD-BM compared to GAPDH, was quantified by qRT-PCR. (**B**) A375 and A2058 cell proliferation was assessed after treatment with and without doxycycline for 48 h. (**C**, **D**) The migration rates of 6×10^4^ A375 cells transfected with genetic circuit plasmids (± doxycycline) were assessed over 36 h by RTCA assay. (***p < 0.001).

To test genetic circuit availability in melanoma cells, we co-transfected constructs into A375 and A2058 cells, and administered doxycycline at final concentration of 1 μM. Quantitative RT-PCR data showed that NVD-BM expression was activated by doxycycline (Figure 7A), and A375 and A2058 proliferation and migration rates were significantly impaired by NVD-BM activation (Figure 7B–7D). Thus, our genetic circuit effectively sensed the dual-input of RELA and STAT1 signals, and induced NVD-BM actuator expression to rebuild cholesterol metabolism by converting cholesterol to 7-DHC, promoting melanoma cell regression.

### NVD-BM functions as a tumor suppressor via Akt1/NF-κB signaling

**Figure 8:**
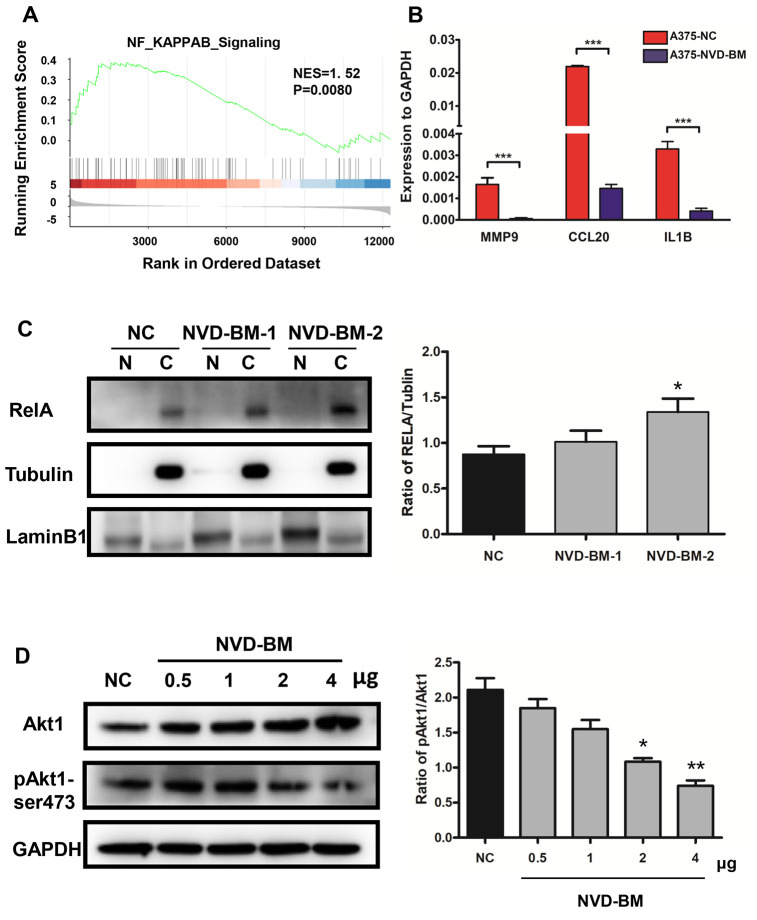
**NVD-BM functions as a tumor suppressor via Akt1/NF-κB signaling.** (**A**) GSEA revealed up-regulated genes associated with NF-κB signaling in melanoma (GSE3189 dataset). (**B**) Gene expression of the NF-κB target genes, MMP9, CCL20 and IL1B relative to GAPDH, in A375 cells transfected with NVD-BM, when compared with A375 cells transfected with blank vector, pCDH. (**C**) Western blot of A375 cells transfected with two different plasmid NVD-BM concentrations, when compared with pCDH blank vectors, analyzing the entry of RELA into the nucleus. (**D**) Western blot of A375 cells transfected with two different plasmid NVD-BM concentrations, when compared to the pCDH blank vector analyzed phosphorylated and unphosphorylated Akt1-SER473 with normalization to GAPDH. (*p < 0.05, **p < 0.01, ***p < 0.001).

Enhanced NF-κB activation, as a result of upstream signaling dysregulation, is a main molecular characteristic in melanoma [38]. Indeed, GSEA analysis of GSE3189 transcriptomic data between normal skin and melanoma samples, demonstrated that NF-κB signaling was up-regulated in melanoma patients (Figure 8A). Thus, we determined the expression of known NF-κB targets in melanoma, e.g., MMP9, CCL20 and IL1B [39, 40]. RT-PCR data demonstrated that MMP9, CCL20 and IL1B expression was significantly down-regulated by NVD-BM (Figure 8B). Similarly, by investigating the cellular localization of free RELA, which is an important transcription factor implicated in NF-κB signaling, we noted that RELA accumulated in the cytoplasm by western blot assay, in a dose dependent NVD-BM manner (Figure 8C), thus, suggesting that RELA translocation to the nucleus was inhibited under these conditions. Next, we determined the phosphorylation status of an upstream NF-κB kinase by western blot assay, and found that Akt1-ser473 phosphorylation was decreased by NVD-BM, in a dose dependent manner (Figure 8D). Collectively, these data indicated that NVD-BM promoted tumor regression by decreasing phosphorylated Akt1 levels, and inhibiting free RELA translocation into the nucleus in melanoma cells.

## DISCUSSION

Metabolic reprogramming has received considerable attention due to its ability to support growth and anti-apoptosis mechanisms in cancers [23]. Disrupting the expression of key enzymes in cholesterol biosynthesis, such as HMGCR and SQLE, appears to prevent carcinogenesis mechanisms in colorectal, breast, prostate and NAFLD-HCC cancers [18–22]. Though comprehensive studies showed that high cellular cholesterol contributes to tumor growth and metastasis, it is still not clear in melanoma yet. Indeed, transcriptome data shows that cholesterol biosynthesis gene activation is associated with poor prognoses in melanoma patients [17], however, the expression of cholesterol biosynthesis limiting enzymes HMGCR was down-regulated in melanoma patients according to analysis of TCGA-SKCM and GSE3189 transcriptome data (Supplementary Figure 2). In present study, administration of 7-DHC *in vitro s*uppressed melanoma cell growth [38], suggesting that the strategic manipulation of cholesterol metabolism may be a potential strategy for melanoma treatment. Because no cholesterol 7-desaturases exist in mammalian cells, we screened and cloned evolutionary homologous cholesterol 7-desaturases from several species and tested them against several melanoma cell pathological characteristics, such as cell proliferation and migration. When compared with DAF-36, NVD-BM from *Bombyx mori* exhibited a stronger ability to inhibit growth in *in vitro* melanoma cells, and growth in an *in vivo* xenograft mouse model. Consistent with GC/MS data, DAF-36 failed to convert cholesterol to 7-dehydrocholesterol. Thus, we believed that NVD-BM is a novel tumor repressor in melanoma.

Synthetic cell-based therapies are ideal approaches for metabolic disorders, as they circumvent the shortcomings of conventional therapies, such as frequent administration and self-sufficient doses. Genetic circuits are typically composed of a sensor, processor and actuator [35]. From our results, NVD-BM was an ideal actuator in promoting 7-DHC accumulation in skin cancer. To ensure specificity, safety and efficacy of the genetic circuit, the sensor and processor were composed of four independent modules, which sensed highly expressed transcription factors in tumors, and activated downstream step by step. The CRISPR-Cas9 system and the Tet-on switch were central to our genetic circuit, guide RNA and Cas9 device were detached on separated module and dimerized once sensing up-regulated transcription factors. RELA and STAT1 binding motifs were blocked, and the switch was activated by doxycycline. Our results showed that RELA and STAT1 activated NVD-BM expression in the presence of doxycycline, and slowed melanoma cell growth and migration. However, endogenous transcription factors usually function as “master regulators”, exerting control over processes that specify cell types and developmental patterning in normal cells. Hence, preventing signal leakage and accurately setting specific threshold values that distinguish tumor and normal cells to trigger circuit activation, should be investigated in the future.

Taken together, we identified NVD-BM from *Bombyx mori* as a novel melanoma tumor suppressor. It decreased Akt1 phosphorylation levels and inhibited RELA translocation into nuclei. We also developed an effective genetic circuit, based on an “AND” gate approach, to activate NVD-BM expression by sensing RELA and STAT1. Our results provide insights to against cancer with cholesterol accumulation by depletion of cholesterol. More importantly, our genetic circuit dual-inputs and actuators are replaceable depend on cancer types, thus providing a general metabolic reprogramming strategy for cancer treatment.

## MATERIALS AND METHODS

### Cell lines and cell culture

A375 malignant melanoma (A375) and 293T embryonic kidney (HEK293T) cells were purchased from Cell Resource Center of Peking Union Medical College (IBMS, CAMS/PUMC). The A2058 melanoma cell line was kindly provided by Dr. Fang from the Beijing Institute of Genomics. All cells were cultured in high-glucose DMEM complete medium (Gibco), with 1% penicillin-streptomycin (Solarbio), and 10% fetal bovine serum (FBS) (Gibco) in a 5% CO_2_, 37°C environment.

### Subcutaneous tumor formation

BALB/c-nu/nu mice, weighing on average 10 g, were purchased from Beijing Huafukang Bioscience Co. Inc. Lentiviral vectors for NVD-BM and pCDH were transfected into A375 cells (n = 4/group), and selected using 1 μg/ml puromycin for two weeks. We then collected 1×10^7^ cells for subcutaneous injection into mice. After four weeks, we measured mouse tumors, and tumor tissues were fixed in 10% neutral buffered formalin for hematoxylin-eosin (HE) staining. The animal protocol was reviewed and approved by the Animal Ethical and Welfare Committee (AEWC) (approval No. IRM-DWLL-2019102).

### Plasmid construction

Codon optimization for cholesterol 7-desaturases from *Caenorhabditis elegans*, *Bombyx mori*, *Danio rerio* (Zebrafish), *Tetrahymena thermophila* SB210 and *Xenopus laevis* were performed by the OptimumGene^TM^ algorithm of GenScript, and then cloned into pCHD-CMV-MCS-EF1-CopGFP-T2A-puro (pCDH) via *EcoRI/BamHI* sites. To select input signals for genetic circuit construction, five repeated motifs from transcription factors were cloned into a firefly luciferase expressed plasmid. All motif sequences are listed (Supplementary Table 2). Furthermore, four genetic circuit plasmids, e.g. pCHD-RELA 5 motif (GGGAATTTCC)-miniP-Cas9-U6P-sgRNA-lacI, pCHD-CMV-lacI, pCHD-STAT1 5 motif (CATTTCCCGGAAACC)-miniP-lacO-rtTA and pCHD-TRE-NVD-BM, were also constructed based on the pCDH plasmid.

### Cell transfection and infection

All cells were transfected with plasmids using Lipofectamine 3000 (Invitrogen) to facilitate DNA transient transfections. For cell infection, viral vectors were transfected into HEK293T cells, after which lentiviruses were collected and concentrated. A375 and A2058 cells were incubated in viral media and polybrene, and incubated in 1 μg/ml puromycin for two weeks.

### Real-time quantitative PCR assay

Total RNA was isolated from cells using Trizol reagent (Invitrogen), and cDNA was generated using the reverse transcription kit (Genecopoeia). Real-time PCR was performed using SYBR PCR mix (TransGen), and GAPDH was used as a reference housekeeping gene. We calculated expression fold changes using the 2^−ΔΔct^ method. All primers for qRT-PCR were designed using Snapgene and Primer 5 software (Supplementary Table 3).

### MTT assay

Cells transfected with different plasmids were cultured in 96-well plates at 5×10^3^ cells/well. After 48 h at 37°C in 5% CO_2_, 10 μl MTT solution was added to each well, and incubated for another 4 h. After this, the medium was removed and 110 μl/well DMSO added. Absorbance was measured at 570 nm on a plate reader (Thermo).

### Real-time cell analysis assay

A375 and A2058 cell migration was analyzed by real-time cell analysis (RTCA). Both cell types were seeded into the upper chamber of an individual CIM-Plate-16 (5×10^3^ cells/well) in 130 μL medium, without FBS. The upper chamber was placed on a lower CIM-Plate-16 chamber, which contained growth medium supplemented with 10% FBS (attractant). Impedance changes resulting from cell migration onto lower membranes were recorded every 5 min, and monitored over 36 h.

### Sterol extraction and analysis

Sterol extraction and analysis were performed as recently described, with some modifications [41]. Approximately 1×10^7^ cells/sample were collected and washed twice in phosphate buffer saline (PBS). Cells were then centrifuged, resuspended in 3 M HCl, and boiled for 3 min. This was performed three times. Cell fragments were collected by centrifugation, and pellets washed in distilled water. Cell precipitates were resuspended in a 2 M NaOH-methanol solution, and incubated at 60°C overnight. After cooling to room temperature, n-hexane was added to extract sterols, after which the n-hexane phase was removed in a vacuum desiccator. Eventually, desiccated sterol products were derived by N-methyl-N-(trimethylsilyl) trifluoroacetamide (MSTFA) at 30°C for 4 h to generate samples for GC-MS analysis.

Sterols were separated on an Agilent 6890 gas chromatograph (GC) (USA), coupled to a Waters time-of-flight mass spectrometer (TOF-MS) (USA). The GC was equipped with a DB-5 fused-silica capillary column (30 m × 0.25 mm internal diameter, film thickness of 0.25 μm (J&W Scientific, CA, USA). Ions were generated by a 70 eV electron beam in EI mode, at an ionization current of 40 μA. Mass spectra were acquired in the 50–800 m/z range. The ion source temperature was 250°C, and the injection site temperature was 260°C. The temperature was initially 70°C for 2 min, then increased by 30°C/min to 250°C, and finally increased to 280°C at 10°C/min. 280°C was maintained for 15 min, and finally increased to 290°C at 5°C/min. The final temperature was maintained for 5 min. Standards (cholesterol and 7-DHC) were purchased from Sigma-Aldrich (USA).

### Western blotting

Protein lysates from cells were lysed in RIPA buffer, with a protease inhibitor cocktail. For western blotting, proteins (20 μg/sample) were separated on 12% SDS-polyacrylamide gel electrophoresis (PAGE) and transferred to nitrocellulose membranes (Millipore). Membranes were blocked in 5% non-fat milk in TBST, and incubated with primary antibodies against Akt1 (dilution, 1:1000; Cat. No. 33224) (Signalway Antibody), pAkt1-ser473 (dilution, 1:1000; Cat. No. 13357) (Signalway Antibody), pAkt1-thr308/309 (dilution, 1:1000; Cat. No. 13311) (Signalway Antibody), LaminB1 (dilution, 1:1000; Cat. No. 40413) (Signalway Antibody), RELA (dilution, 1:1000; Cat. No. ab19870) (Abcam), Tubulin (dilution, 1:5000; Cat. No. Z0305) (Ray Antibody), and GAPDH (dilution, 1:5000; Cat. No. ab181602) (Abcam). After washing, membranes were incubated with anti-mouse (dilution, 1:5000; Cat. No. ab97040) (Abcam), or anti-rabbit (dilution, 1:5000; Cat. No. ab7090) (Abcam) peroxidase-conjugated secondary antibodies. Protein bands were revealed by ECL reagent on Exposure meter.

### Luciferase assay

Cells were co-transfected with firefly luciferase and Renilla luciferase plasmids for 48 h. Growth medium was then removed, and monolayers washed gently and thoroughly in PBS, to avoid disruption. PBS was complexly removed. Then 1×PLB (Promega) was added to each well, and plates rocked at room temperature for 15 min. 100 μl LAR II (Promega) was added to luminometer tubes, including 20 μl cell lysates. Absorbance was measured at 560 nm. 100 μl Stop & Glo reagents (Promega) were then added to each tube, and absorbance measured at 560 nm. Luciferase results were determined by dividing absorbance at 560 nm by absorbance at 470 nm.

### Bioinformatics analysis

R software was used to analyze bioinformatics data from GEO (https://www.ncbi.nlm.nih.gov/gds/), TCGA (https://www.cancer.gov/about-nci/organization/ccg/research/structural-enomics/tcga) and GTEX (http://genome.ucsc.edu/gtex.html) databases. Firstly, we downloaded transcriptome data from melanoma patient samples from the TCGA database, and normal skin sample data from the GTEX database, which are skin cutaneous melanoma (813 normal tissues, 471 tumor tissues). Then we downloaded one GEO dataset, GSE3189, i.e. transcriptome melanoma data comprising 7 normal and 45 melanoma tissues. Secondly, to compare gene expression between normal and tumor samples, we normalized RNA-Seq data from these public databases, and used the limma R package to analyze gene expression patterns, to screen significantly differentially expressed genes (log Fold Change > 1, adj-p value < 0.05). The clusterProfiler R package was used for Gene Set Enrichment Analyses (GSEA). In addition, statistical significance parameters (p value) for all data were calculated by T tests.

## Supplementary Material

Supplementary Figures

Supplementary Table 1

Supplementary Tables 2 and 3

## References

[1] Schadendorf D, van Akkooi AC, Berking C, Griewank KG, Gutzmer R, Hauschild A, Stang A, Roesch A, Ugurel S. Melanoma. Lancet. 2018; 392:971–84. 10.1016/S0140-6736(18)31559-9 30238891

[2] Olazagasti Lourido JM, Ma JE, Lohse CM, Brewer JD. Increasing incidence of melanoma in the elderly: an epidemiological study in olmsted county, minnesota. Mayo Clin Proc. 2016; 91:1555–62. 10.1016/j.mayocp.2016.06.028 27692970 PMC5118041

[3] Crocetti E, Mallone S, Robsahm TE, Gavin A, Agius D, Ardanaz E, Lopez MC, Innos K, Minicozzi P, Borgognoni L, Pierannunzio D, Eisemann N, and EUROCARE-5 Working Group. Survival of patients with skin melanoma in europe increases further: results of the EUROCARE-5 study. Eur J Cancer. 2015; 51:2179–90. 10.1016/j.ejca.2015.07.039 26421821

[4] Menzies AM, Long GV. Systemic treatment for BRAF-mutant melanoma: where do we go next? Lancet Oncol. 2014; 15:e371–81. 10.1016/S1470-2045(14)70072-5 25079100

[5] Ascierto PA, Ferrucci PF, Fisher R, Del Vecchio M, Atkinson V, Schmidt H, Schachter J, Queirolo P, Long GV, Di Giacomo AM, Svane IM, Lotem M, Bar-Sela G, et al. Dabrafenib, trametinib and pembrolizumab or placebo in BRAF-mutant melanoma. Nat Med. 2019; 25:941–46. 10.1038/s41591-019-0448-9 31171878

[6] Hauschild A, Grob JJ, Demidov LV, Jouary T, Gutzmer R, Millward M, Rutkowski P, Blank CU, Miller WH Jr, Kaempgen E, Martín-Algarra S, Karaszewska B, Mauch C, et al. Dabrafenib in BRAF-mutated metastatic melanoma: a multicentre, open-label, phase 3 randomised controlled trial. Lancet. 2012; 380:358–65. 10.1016/S0140-6736(12)60868-X 22735384

[7] Robert C, Grob JJ, Stroyakovskiy D, Karaszewska B, Hauschild A, Levchenko E, Chiarion Sileni V, Schachter J, Garbe C, Bondarenko I, Gogas H, Mandalá M, Haanen JB, et al. Five-year outcomes with dabrafenib plus trametinib in metastatic melanoma. N Engl J Med. 2019; 381:626–36. 10.1056/NEJMoa1904059 31166680

[8] Ascierto PA, McArthur GA, Dréno B, Atkinson V, Liszkay G, Di Giacomo AM, Mandalà M, Demidov L, Stroyakovskiy D, Thomas L, de la Cruz-Merino L, Dutriaux C, Garbe C, et al. Cobimetinib combined with vemurafenib in advanced BRAF(V600)-mutant melanoma (coBRIM): updated efficacy results from a randomised, double-blind, phase 3 trial. Lancet Oncol. 2016; 17:1248–60. 10.1016/S1470-2045(16)30122-X 27480103

[9] Sullivan RJ, Hamid O, Gonzalez R, Infante JR, Patel MR, Hodi FS, Lewis KD, Tawbi HA, Hernandez G, Wongchenko MJ, Chang Y, Roberts L, Ballinger M, et al. Atezolizumab plus cobimetinib and vemurafenib in BRAF-mutated melanoma patients. Nat Med. 2019; 25:929–35. 10.1038/s41591-019-0474-7 31171876

[10] Das Thakur M, Salangsang F, Landman AS, Sellers WR, Pryer NK, Levesque MP, Dummer R, McMahon M, Stuart DD. Modelling vemurafenib resistance in melanoma reveals a strategy to forestall drug resistance. Nature. 2013; 494:251–55. 10.1038/nature11814 23302800 PMC3930354

[11] Helmbach H, Rossmann E, Kern MA, Schadendorf D. Drug-resistance in human melanoma. Int J Cancer. 2001; 93:617–22. 10.1002/ijc.1378 11477569

[12] Morandi A, Indraccolo S. Linking metabolic reprogramming to therapy resistance in cancer. Biochim Biophys Acta Rev Cancer. 2017; 1868:1–6. 10.1016/j.bbcan.2016.12.004 28065746

[13] Simons K, Ikonen E. How cells handle cholesterol. Science. 2000; 290:1721–26. 10.1126/science.290.5497.1721 11099405

[14] Tall AR, Costet P, Wang N. Regulation and mechanisms of macrophage cholesterol efflux. J Clin Invest. 2002; 110:899–904. 10.1172/JCI16391 12370265 PMC151157

[15] Tabas I. Consequences of cellular cholesterol accumulation: basic concepts and physiological implications. J Clin Invest. 2002; 110:905–11. 10.1172/JCI16452 12370266 PMC151158

[16] Cruz PM, Mo H, McConathy WJ, Sabnis N, Lacko AG. The role of cholesterol metabolism and cholesterol transport in carcinogenesis: a review of scientific findings, relevant to future cancer therapeutics. Front Pharmacol. 2013; 4:119. 10.3389/fphar.2013.00119 24093019 PMC3782849

[17] Kuzu OF, Noory MA, Robertson GP. The role of cholesterol in cancer. Cancer Res. 2016; 76:2063–70. 10.1158/0008-5472.CAN-15-2613 27197250 PMC5813477

[18] Jacobs EJ, Rodriguez C, Brady KA, Connell CJ, Thun MJ, Calle EE. Cholesterol-lowering drugs and colorectal cancer incidence in a large united states cohort. J Natl Cancer Inst. 2006; 98:69–72. 10.1093/jnci/djj006 16391373

[19] Wang C, Li P, Xuan J, Zhu C, Liu J, Shan L, Du Q, Ren Y, Ye J. Cholesterol enhances colorectal cancer progression via ROS elevation and MAPK signaling pathway activation. Cell Physiol Biochem. 2017; 42:729–42. 10.1159/000477890 28618417

[20] Borgquist S, Bjarnadottir O, Kimbung S, Ahern TP. Statins: a role in breast cancer therapy? J Intern Med. 2018; 284:346–57. 10.1111/joim.12806 29923256 PMC6175478

[21] Stopsack KH, Gerke TA, Sinnott JA, Penney KL, Tyekucheva S, Sesso HD, Andersson SO, Andrén O, Cerhan JR, Giovannucci EL, Mucci LA, Rider JR. Cholesterol metabolism and prostate cancer lethality. Cancer Res. 2016; 76:4785–90. 10.1158/0008-5472.CAN-16-0903 27325648 PMC4987257

[22] Liu D, Wong CC, Fu L, Chen H, Zhao L, Li C, Zhou Y, Zhang Y, Xu W, Yang Y, Wu B, Cheng G, Lai PB, et al. Squalene epoxidase drives NAFLD-induced hepatocellular carcinoma and is a pharmaceutical target. Sci Transl Med. 2018; 10:eaap9840. 10.1126/scitranslmed.aap9840 29669855

[23] Silvente-Poirot S, Poirot M. Cancer. Cholesterol and cancer, in the balance. Science. 2014; 343:1445–46. 10.1126/science.1252787 24675946

[24] Swinnen JV, Brusselmans K, Verhoeven G. Increased lipogenesis in cancer cells: new players, novel targets. Curr Opin Clin Nutr Metab Care. 2006; 9:358–65. 10.1097/01.mco.0000232894.28674.30 16778563

[25] McDonnell DP, Park S, Goulet MT, Jasper J, Wardell SE, Chang CY, Norris JD, Guyton JR, Nelson ER. Obesity, cholesterol metabolism, and breast cancer pathogenesis. Cancer Res. 2014; 74:4976–82. 10.1158/0008-5472.CAN-14-1756 25060521 PMC4167494

[26] Wu Q, Ishikawa T, Sirianni R, Tang H, McDonald JG, Yuhanna IS, Thompson B, Girard L, Mineo C, Brekken RA, Umetani M, Euhus DM, Xie Y, Shaul PW. 27-hydroxycholesterol promotes cell-autonomous, ER-positive breast cancer growth. Cell Rep. 2013; 5:637–45. 10.1016/j.celrep.2013.10.006 24210818 PMC3950897

[27] Bauriaud-Mallet M, Vija-Racaru L, Brillouet S, Mallinger A, de Medina P, Rives A, Payre B, Poirot M, Courbon F, Silvente-Poirot S. The cholesterol-derived metabolite dendrogenin A functionally reprograms breast adenocarcinoma and undifferentiated thyroid cancer cells. J Steroid Biochem Mol Biol. 2019; 192:105390. 10.1016/j.jsbmb.2019.105390 31170473

[28] Lin CY, Huo C, Kuo LK, Hiipakka RA, Jones RB, Lin HP, Hung Y, Su LC, Tseng JC, Kuo YY, Wang YL, Fukui Y, Kao YH, et al. Cholestane-3β, 5α, 6β-triol suppresses proliferation, migration, and invasion of human prostate cancer cells. PLoS One. 2013; 8:e65734. 10.1371/journal.pone.0065734 23785446 PMC3681800

[29] de Medina P, Paillasse MR, Segala G, Voisin M, Mhamdi L, Dalenc F, Lacroix-Triki M, Filleron T, Pont F, Saati TA, Morisseau C, Hammock BD, Silvente-Poirot S, Poirot M. Dendrogenin a arises from cholesterol and histamine metabolism and shows cell differentiation and anti-tumour properties. Nat Commun. 2013; 4:1840. 10.1038/ncomms2835 23673625 PMC3674249

[30] Gelzo M, Granato G, Albano F, Arcucci A, Dello Russo A, De Vendittis E, Ruocco MR, Corso G. Evaluation of cytotoxic effects of 7-dehydrocholesterol on melanoma cells. Free Radic Biol Med. 2014; 70:129–40. 10.1016/j.freeradbiomed.2014.02.013 24561580

[31] Yoshiyama-Yanagawa T, Enya S, Shimada-Niwa Y, Yaguchi S, Haramoto Y, Matsuya T, Shiomi K, Sasakura Y, Takahashi S, Asashima M, Kataoka H, Niwa R. The conserved rieske oxygenase DAF-36/neverland is a novel cholesterol-metabolizing enzyme. J Biol Chem. 2011; 286:25756–62. 10.1074/jbc.M111.244384 21632547 PMC3138242

[32] Yoshiyama T, Namiki T, Mita K, Kataoka H, Niwa R. Neverland is an evolutionally conserved rieske-domain protein that is essential for ecdysone synthesis and insect growth. Development. 2006; 133:2565–74. 10.1242/dev.02428 16763204

[33] Najle SR, Nusblat AD, Nudel CB, Uttaro AD. The sterol-C7 desaturase from the ciliate tetrahymena thermophila is a rieske oxygenase, which is highly conserved in animals. Mol Biol Evol. 2013; 30:1630–43. 10.1093/molbev/mst076 23603937

[34] Zhang T, Bao J, Zhang M, Ge Y, Wei J, Li Y, Wang W, Li M, Jin Y. Chemo-photodynamic therapy by pulmonary delivery of gefitinib nanoparticles and 5-aminolevulinic acid for treatment of primary lung cancer of rats. Photodiagnosis Photodyn Ther. 2020; 101807. 10.1016/j.pdpdt.2020.101807 32404298

[35] Brophy JA, Voigt CA. Principles of genetic circuit design. Nat Methods. 2014; 11:508–20. 10.1038/nmeth.2926 24781324 PMC4230274

[36] Lienert F, Lohmueller JJ, Garg A, Silver PA. Synthetic biology in mammalian cells: next generation research tools and therapeutics. Nat Rev Mol Cell Biol. 2014; 15:95–107. 10.1038/nrm3738 24434884 PMC4032074

[37] Teixeira AP, Fussenegger M. Synthetic biology-inspired therapies for metabolic diseases. Curr Opin Biotechnol. 2017; 47:59–66. 10.1016/j.copbio.2017.06.004 28662442

[38] De Donatis GM, Le Pape E, Pierron A, Cheli Y, Hofman V, Hofman P, Allegra M, Zahaf K, Bahadoran P, Rocchi S, Bertolotto C, Ballotti R, Passeron T. NF-kB2 induces senescence bypass in melanoma via a direct transcriptional activation of EZH2. Oncogene. 2016; 35:2813. 10.1038/onc.2015.468 27225420

[39] Cheng Q, Wu J, Zhang Y, Liu X, Xu N, Zuo F, Xu J. SOX4 promotes melanoma cell migration and invasion though the activation of the NF-κB signaling pathway. Int J Mol Med. 2017; 40:447–53. 10.3892/ijmm.2017.3030 28627651 PMC5504990

[40] Gallagher SJ, Mijatov B, Gunatilake D, Gowrishankar K, Tiffen J, James W, Jin L, Pupo G, Cullinane C, McArthur GA, Tummino PJ, Rizos H, Hersey P. Control of NF-kB activity in human melanoma by bromodomain and extra-terminal protein inhibitor I-BET151. Pigment Cell Melanoma Res. 2014; 27:1126–37. 10.1111/pcmr.12282 24924589

[41] Guo XJ, Xiao WH, Wang Y, Yao MD, Zeng BX, Liu H, Zhao GR, Yuan YJ. Metabolic engineering of Saccharomyces cerevisiae for 7-dehydrocholesterol overproduction. Biotechnol Biofuels. 2018; 11:192. 10.1186/s13068-018-1194-9 30026807 PMC6047132

