# Supplementary material for: NVD-BM-mediated genetic biosensor triggers accumulation of 7-dehydrocholesterol and inhibits melanoma via Akt1/NF-ĸB signaling
Source: Aging (Albany NY). 2020 Jul 25;12(14):15021–36. doi: 10.18632/aging.103562 (PMC7425431; doi:10.18632/aging.103562)
Supplement: Supplementary Figures [file aging-12-103562-s003..pdf]

SUPPLEMENTARY FIGURES

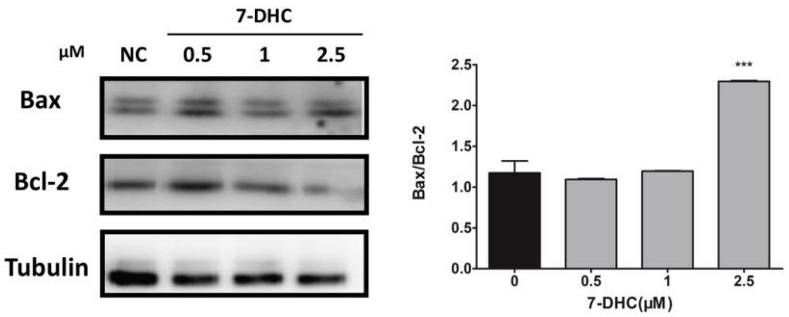

Supplementary Figure 1. Western blot of A375 cells added different concentration of 7-DHC invitro, when compared to the Bax and Bcl2 expression level to detect the capable of inducing melanoma cells apoptosis of 7-DHC with normalization of Tublin. (***p < 0.001).

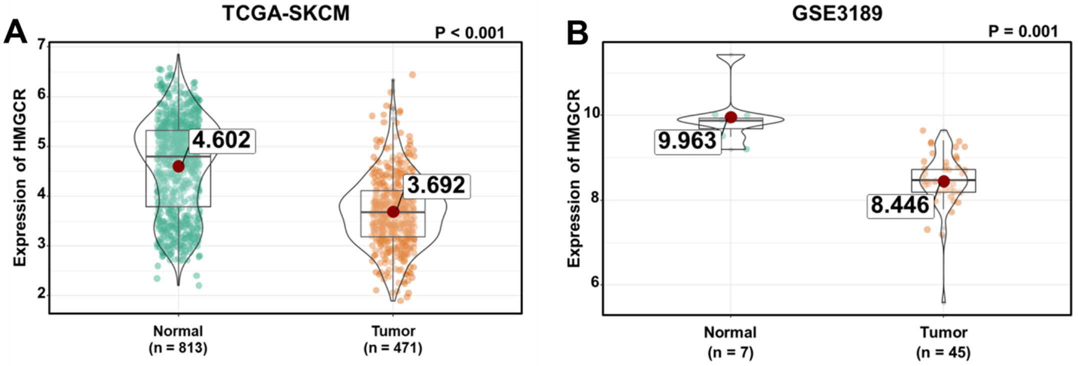

Supplementary Figure 2. The expression of cholesterol biosynthesis limiting enzymes HMGCR was down-regulated in melanoma patients. (**A**) The expression of HMGCR in melanoma and normal skin tissues by differential expression analysis of TCGA-SKCM transcriptome data. (**B**) The expression of HMGCR in melanoma and normal skin tissues by differential expression analysis of GSE3189 transcriptome data.
