# Supplementary material for: NVD-BM-mediated genetic biosensor triggers accumulation of 7-dehydrocholesterol and inhibits melanoma via Akt1/NF-ĸB signaling
Source: Aging (Albany NY). 2020 Jul 25;12(14):15021–36. doi: 10.18632/aging.103562 (PMC7425431; doi:10.18632/aging.103562)
Supplement: Supplementary Table 1 [file aging-12-103562-s001..docx]

**Supplementary Table 1.** **The Codon-optimized sequences of heterogeneous cholesterol 7-desaturase.**

| Gene Symbol | ORF Sequences (5’-3’) |
| --- | --- |
| NVD-BM | ATGGCCGACAGACAGCACTTCCCAAGCGCCATCACCGAGGCCGTGAGCTCCAACACAGCATGCCCAGATACCGGACCTAAGGCAGAGACCACAAACATCTTCCTGCTGCTGCAGAGGAATATCACCATCGAGTCTAGCAAGCACGTGTTTTCCTCTATCGTGGAGTATATCCTGATCCTGACACTGATGTTCGCCTTTTCCGCCATCCTGTACGTGATCTATAAGAGCTACATCTCCCCAGTGTTCTACAAGAAGGAGCTGACCGAAGTGGGCTTTGACCACATCCCACAGGGCCCCGATAAGGGCCGGAGAATCTCTCGGGCACAGGCCAGCAGGAGGATGGGCTCCAAGCTGCCACCTCCATACCCCAACGGCTGGTTTGCCGTGGCCGAGACAAGAGAGCTGAAGGTCGGCTCTGCCCTGAGCATCGACGCACTGGGCCAGAACCTGTGCGTGTACAGGGGAGAGGACGGCCTGGCCAGATGCGTGGATGCCTACTGTCCTCACCTGGGAGCAAACCTGGCAGTGGGAGGAACCGTGAGGGGCTCCTGCATCGAGTGTCCATTCCACAAGTGGCGCTTTAATGCAGCAGGAACATGCGTGAGCCTGCCTGGCTCCGACATCGCACCAAAGGGCGTGTCTATCAGGACATGGTGCGTGGTGGAGACCGACGGAGCCGTGTGGATCTGGCACGATGCAGAGGGAAGGGAGCCTCTGTGGGAGATCACCGATCCCCCTGAGCTGAAGGAGTTCGGCTATCGGGGCAGAAACGAGTTTGAGGTGTCTGCCCACATCCAGGAGATCCCAGAGAACGGAGCAGACGTGCCTCACCTGAATGCCGTGCACAGCTCCTCTCTGCTGAGCGATCTGGGAGAGCGGTACCCCGTGCTGCACGAGATCATCGGCAGACACGTGTGGAATGCCGATTGGACAAAGTCTGACGATCACACCAGCCTGATGCACATCACACAGGAGTATAAGGTGCTGAAGTACGACCTGGCCCGGATCGATGTGAAGGTGACCCAGATCGGCCCAGGCCACGTGAGACTGTTCCTGAAGACCAGCGTGGGCCCCTTTTATATCGTGCAGTCCGTGACACCTCTGGGCCCACTGCTGCAGAAAGTGATCCACAGGGTGTATAGCCCAGCATACAACGCACCAGTGGGAGCCTTCCTGGTGAGGTGCGAGGCCTACATGTTTGAGCGCGACGTGACAATCTGGAACAGCAAGCGGTTCGTGAGCGCCCCCGCCTATGTGAAGACCGATAAGACAATCAGGACCTTCCGCAACTGGTTCGGCCAGTTTTACTCCGAGCACTCTCTGGGCTTTCGGGACGCCCTGCAGAATCCTCTGGATTGGTGA |
| DAF-36 | ATGCTGCTGGAGCAGATCTGGGGCTTCCTGACCGCCCACCCTATCAGCGTGGTGACCACAATCCTGATCGTGTACCTGATCCACATCACACTGAAGCCACTGAATCGCGTGCGGAGACTGGGCGACGTGGGACTGTTCTTTGGCAAGCCCGAGCTGAAGGGCTTCTACAGGGAGAGGCAGCTGGAGCGGCTGAAGCTGCTGAGGCGCGTGGGCGATATGCCACCCGTGTTCCCAAACGGCTGGTATTGCGTGTGCGAGAGCGAGAAGCTGGCCAACAATCAGATCATGGAGATCACCGTGCTGGGCCAGTTCCTGTCCCTGATCCGCTCTGAGAGCGGCGCCGTGTACATCACAGACTCTTATTGCCCCCACATCGGCGCCAACTTCAACATCGGCGGCAGAGTGGTGCGGGATAATTGCATCCAGTGTCCTTTCCACGGCTGGATCTTTTCCGCCGAGACCGGCAAGTGCGTGGAGGTGCCATATGACGAGGGCAGAATCCCCGAGCAGGCCAAGGTGACCACATGGCCATGCATCGAGAGGAACAATAACATCTACCTGTGGTATCACTGTGACGGAGCAGAGCCAGAGTGGGAGATCCCTGAGATCACCGAGATCACAGATGGCTTCTGGCACCTGGGCGGCAGAACCGAGCACGAAGTGATGTGCCACATCCAGGAGATCCCCGAGAATGGCGCCGACATCGCCCACCTGAACTACCTGCACAAGTCCGCCCCACCCGTGACCAAGGGCTCTGATATCATCAAGACAGACCTGAGCGATCCTCAGCCAGCAGTGCAGCACGTGTGGGACGGCAAGTGGGAGGTGAAGTCTGAGGAGGATAGGCACTGTGGCGTGATGCACCTGAATCAGTTCATGACCTTTTGGGGCTATAAGGTGCCCCTGACAAGCTCCAAGCTGGTGGCAGAGCAGCACGGACCTGGCATCGTGCACATGCTGTTCGATTTTGGCATCTGGGGCAAGGGCGTGGTGTTCCAGACCGTGACACCTGAGGAGGCCCTGCTGCAGCGGGTGAGATTCAGGATCTTTAGCAACATCCCATGGTTCTTTGTGAAGTTCTTTATGACCGTGGAGGCCATGCAGTTCGAGCGGGACGTGTTTATCTGGAGCAATAAGAAGTACATCAAGTCCCCTCTGCTGGTGAAGAACGATGGCCCAATCCAGAAGCACCGGAGATGGTTCTCTCAGTTTTATACAGAGAATAGCCCCAAGATGCTGAAGGACGGCTCCCTGTCTAACCAGGCCAAGTCCATCTTTGATTGGTGA |
| NVD-DR | ATGGAGAACACCCGGGCCTCCCTGATGTTCAAGACACTGGCCGTGGCAGCAATCGGACTGAGCGCCACCTTCGTGATGCTGGTGAGAGACCCTTCCGATACACTGTTTGGAGGAGGATACCCAGAGCTGTGGAGGAGAACCGGCCTGGCCGGAGCACCAACAAGGGCTGCCGCCTGCATCTTCGCAGGCGTGTTTCTGCTGGCCATGGGCTGGCTGTACAGGCTGCTGTTTGCACCCCTGGAGCTGCTGAGGGGAGTGGACGAAGTGGGCTATATCGCAGAGGATGGCCGGAGCCGGGCACAGGCCGCCAACGAGGTGAGGCGCCGGAGAAAGACCGGAGAGCTGCCACCCGTGTACCCTAATGGCTGGTATAGGGTGCTGGACTCCCACATGCTGGAGCGCGGCGATGTGAAGTCTGTGACAGTGCTGGGACAGCAGGTGGCCGTGTTCAGGGGACAGGACGGCAAGGCATACGTGGTGGATGCCTATTGTCCTCACCTGGGAGCAAACCTGGCAGTGGGCGGCAGAGTGGTGGGCGGCTGCATCGAGTGTCCATTCCACGGATGGCAGTTTAGGGGAGTGGACGGCCGCTGCGTGAAGATCCCATATGCCGATAAGGTGCCAGAGTTCGCAAAGGTGCGGTGCTGGCCCAGCTGTGAGATCAATGGCCTGGTGCTGGTGTGGTTTCACTGTGACGGCCTGGAGCCATCCTGGAGGGTGCCTGAGCAGTCTCAGATCACCAGAGGCGAGTGGGTGTACAGGGGCCGCACAGAGCACTTTATCAACGCCCACATCGAGGAGATCCCAGAGAATGCAGCAGATATCGCACACCTGGCACACCTGCACACCCCAGGAATCGTGAGCGGAGTGGACCTGCGGTATACCAACTCCAAGACATGGGAGTTCATCAGACACGATTGGAAGGTGGAGTGGAAGCCTGAGCCAGAGCCCAATAAGCACTGCTCCCAGATGCTGGTGAAGCACGCCCTGACCGTGTTTGGCAGGCACTGGCCACTGCTGGACCTGGATGTGCTGGCCAGGCAAGTGGGACCTGGAGTGGTGTTCCTGCTGTTTGAGCACTCTTTCCTGGGCAGAGGCGTGATCATGCACTGCGTGACCCCTGTGGAGCCACTGCTGCAGTGCGTGAGCCACACAATCTTTTACCAGAGCTCCATCCCACCCCTGGTGCCCAAGTTCATCCTGCGGGCCGAGTGTATCCAGTTTGAGAGAGACGTGATGATCTGGAACAATAAGACCTATATCTCTAAGCCCATGCTGGTGAAGGAGGATAGCGCCATCCAGAAGCACAGGCGCTGGTTCAGCCAGTTTTACTCTGAGAATAGCCCTCGGCTGAGATATCAGCACGACACACTGGATTTCTGA |
| TTHERM_00310640 | ATGATCGAGTTCAACAAGGAGTGCCTGATGGATATCCTGAAGAATCAGGACTACCACTTCTATATGGTCATCCCCCTGATCTTTATCGGCCTGTACGCCCTGTACATCAAGAAGTTTAAGTACTACAACCCTATCGAGAAGCAGGAGTGGGACGATCGGAGATCCAACGTGAAGCGGGGCAATCCCCCTCCATCTTACCCTAACGGCTGGTTCAGAGTGTGCCACAAGAATGAGCTGCAGATCGGCCAGTCTAAGTTCTTTAAGCTGAATGGCCGGCACATCACCGTGTTTAGAGGAGAGGACGGCATCCCCTACGCACTGCACGCCTATTGTAGCCACATGGGAGCAAACCTGGGAATCGGAGGCAAGGTGAAGTGGAACAGCTGCATCGAGTGTCCTTTCCACGGCTGGAGCTTTGACGGCAAGTCCGGCAAGTGCGTGAACTCCGAGCACCTGGATGAGAAGCAGTGTACCCACCACACATACCACGATATCAAGAAGATGACCAAGGGCTCTGACAATAGGTATATCAAGACATGCGAGAGCGGCTCCCCCTCTCAGATCCAGAAGTTCCACGTGCGCCAGCAGAACAATCTGATCTACGTGTGGTTCCACGCCAAGAACGTGGACCCCTACTATGAGCCTTTTGAGATCAATGAGATCCCATACCTGGAGGATCGGGGCGAGACCGCCGACTATGTGAACTGTCAGATCCAGGAGATCCCCGAGAATGGCGCCGACTTCAAGCACTTTGAGTACGTGCACTATGCCTGGATCGAGATCCTGTTCCCTTGGATCAAGTTTAAGTGGGTGCCAAAGGATAGGAAGCCCACCGACAAGGATTTCGACGAAGTGATGCGCACACACCCAAACAAGAAGGTGCAGGCCTTCAGCAATAAGCTGTTTGATAAGTACACCAACGAGCAGAATAAGACACACATCAACAATCTGGTGCTGGATGCCTACCTGGTGTTCTTTGACAAGTTCGAGTTTTATATCCAGACCGCCACAGTGTTCCAGCTGGGCTCTGGCACCGTGTTCCTGTTTCTGAAGTTTCCTCTGTGGGAGGCAGTGGTGGTGCAGAGCGTGACCCCAGTGGGCAAGTTCAACCAGCTGGTGCACCACAAGATGTATACATCTTGGTGGCTGCCATACTGGGTGAGCGCCTATCTGCTGGCCGGCTTCAGAAAGCAGTTTATCAGCGATAAGATCGTGTGGAACAATAAGATCTTCGCCGACAAGCTGACATACAACCCCAAGGCCGTGTTTGATGAGAGGCTGCTGAATTGGCGCGAGTGGTATTCCCAGTACTATGAGGGCTGTGATGAGTTCGAGAAGAACCAGGAGGCCTTTGACTGGTGA |
| MGC154819 | ATGGAGTCCGTGGGCCACTCTCTGCTGACACTGTCTGTGCTGTGCCCAGTGGGCGCCCTGCTGCTGTGCTGGGTGGGCAGCGTGCTGCTGGGAGCAGGACTGCTGCCAAGCCTGACCCTGTCCACAAGAGACCCCTCTACCACACTGAGCAGGACCCCATGGCTGGTGGTGCTGGTGCCTCTGCTGGTGCTGTGGGGCTGGAGATGGCTGAGCAGGCCAATGGAGCTGCTGCGGTCCCCAGAGGAAGTGGGATACATCCCTGAGAGGGGCCGCTCTCGGGCACAGACAGCCAACCTGGTGCGGAGAAGGCGCATGAAGGGCGAGCTGCCCCCTATCTACCCCAATGGCTGGTATAGAGCCCTGGACAGCCACCTGCTGCCACCAGGAACCGTGCAGGATTGCACACTGCTGGGCGAGCAGCTGGCCGTGTACAGGACCCTGGAGGGCAAGGTGTACGTGGTGGACGCCTATTGTCCACACCTGGGAGCAAACCTGGCAGTGGGAGGCAAGGTGGTGGGCGATTGCATCGAGTGTCCCTTCCACGGATGGCAGTTTCGCGGAGAGGACGGCAAGTGCACAAGAATCCCCTACGCCGAGAAGGTGCCAGATTTCGCCAAGATCAAGACCCGCCCTAGCTGCGAGCTGAATGGCATGGTGTTCGTGTGGTATCACTGTGACGGCATCGAGCCAACCTGGTCCGTGCCCGAGCAGGAGGAGATCACAAAGAAGGAGTGGGTGTACCACGGCCGGACCGAGCACTATGTGAACGCCCACATCGAGGAGATCCCCGAGAATGCCGCCGACATCGCCCACCTGGATTTCCTGCACACACCTGGCATCCTGTCTGGCGTGGACCTGCGCTACACCAAGAGCCGGATCTGGGATTTTGTGAAGCACTCCTGGAAGGTGCAGTGGATTCCCGAGCCAGCCCCCTATAAGCACTGTTCCCAGATGCTGCTGGCCCACTCTATCCTGCTGTTCGGCAAGCACTTTCCTCTGCTGGATGTGAACGTGGTGGCCAGACAAGTGGGCCCAGGCATCGTGTTCCTGCACTTTAAGCACGCCTTCCTGGGCGAGGGCGTGATCGTGCACTGCGTGACACCTGTGGAGCCACTGCTGCAGAAGGTGAGCCACTCCATCTACTATCAGAAGAATATCCCCGCCCTGATCCCTAAGTTCATCCTGAAGGCCGAGTGTATCCAGTTTGAGAGGGACGTGATGATCTGGAACAATAAGAAGTACATCTCCAAGCCTATGCTGGTGAAGGAGGATGCCGCCATCCAGAAGCACCGGAGATGGTTCTCTCAGTTTTATTCTAACAATAGCCCACAGATCACCTTTCAGCAGGAGGGCCTGGATTGGTGA |
