# Supplementary material for: NVD-BM-mediated genetic biosensor triggers accumulation of 7-dehydrocholesterol and inhibits melanoma via Akt1/NF-ĸB signaling
Source: Aging (Albany NY). 2020 Jul 25;12(14):15021–36. doi: 10.18632/aging.103562 (PMC7425431; doi:10.18632/aging.103562)
Supplement: Supplementary Tables 2 and 3 [file aging-12-103562-s002..pdf]

SUPPLEMENTARY TABLES

Supplementary Table 2. Motifs of top 10 highly expressed transcription factors in melanoma.

Transcription factors	Motif sequences (5’-3’)
RELA	GGGAATTTCC
STAT1	CATTTCCCGGAAACC
FOXD1	GTAAACAT
POU3F4	TATGCAAAT
SOX5	TTAACAATAC
ELF1	GAACCAGGAAGTG
TBX2	AAGGTGTGAAA
LEF1	AAAGATCAAAGGGTT
HOXD13	CCAATAAAAA
POU3F2	TTATGCAAATTA

Supplementary Table 3. Primers of qRT-PCR in this study.

Primers	Sequences (5’-3’)
GAPDH_F	AGCCACATCGCTCAGACAC
GAPDH_R	GCCCAATACGACCAAATCC
NVD-BM_F	ACGGACTGCACGATATAAAAGGG
NVD-BM_R	ATCACACCAGCCTGATGCAC
DAF-36_F	AGTGCGTGGAGGTGCCATA
DAF-36_R	TGGCTCTGCTCCGTCACAGTGA
NVD-DR_F	TGGATGCCTATTGTCCTCACCTGG
NVD-DR_R	TCCACTCCCCTAAACTGCCATCC
TTHEM-00310640_F	AAGCAGTGTACCCACCACACATAC
TTHEM-00310640_R	TTCTGCTGGCGCACGTGGAA
MGC154819_F	ATTGCATCGAGTGTCCCTTCCAC
MGC154819_R	TCTTGGCGAAATCTGGCACCTTC
MMP9_F	AGTCCACCCTTGTGCTCTTC
MMP9_R	ACTCTCCACGCATCTCTGC
CCL20_F	TGGATACACAGACCGTATTCTTCATCC
CCL20_R	AGTCTGTTTTGGATTTGCGCAC
IL1B_F	AGGAGAATGACCTGAGCACCTTC
IL1B_R	ATCGTACAGGTGCATCGTGC
